# The effect of high-fat diet on the pharmacokinetics of ondansetron hydrochloride tablets in healthy Chinese subjects

**DOI:** 10.3389/fphar.2025.1512857

**Published:** 2025-03-21

**Authors:** Na Zhao, Caiyun Jia, Yiting Hu, Xue Sun, Haojing Song, Bo Qiu, Wanjun Bai, Zhanjun Dong

**Affiliations:** Department of Pharmacy, Hebei General Hospital, Hebei Key Laboratory of Clinical Pharmacy, Shijiazhuang, China

**Keywords:** food, high-fat diet, ondansetron, safety, pharmacokinetics

## Abstract

**Background:**

This study aimed to assess how a high-fat diet impacts the pharmacokinetics and safety characteristics of 8 mg Ondansetron hydrochloride tablets among healthy Chinese individuals.

**Subjects and methods:**

The findings presented here were obtained from a bioequivalence study, in which individuals were randomly assigned to consume Ondansetron hydrochloride tablets either following a meal or subsequent to a high-fat diet containing 978.6 kcal, with 54.6% of the calories derived from fat. The plasma concentrations of Ondansetron were measured through the utilization of high-performance liquid chromatography-mass spectrometry (LC-MS/MS) after collecting blood samples. For the computation of pharmacokinetic parameters, the non-compartmental module from Phoenix WinNonlin Version 8.2 was utilized Additionally, the BE module within WinNonLin was utilized to statistically analyze key pharmacokinetic metrics, including the maximum level of concentration (Cmax), the area beneath the concentration-time curve spanning from zero to the final quantifiable time point (AUC_0-t_), and the area beneath the concentration-time curve extending from zero to a theoretical limitless point (AUC_0–∞_) in plasma. A total of 53 healthy subjects participated in the study and were divided into a fasted cohort and a postprandial cohort.

**Results:**

Ondansetron had lower Cmax, AUC_0–t_, and AUC_0–∞_in plasma when taken with food compared to when taken on an empty stomach, with the 90% confidence interval falling outside the acceptable range of 80.00%–125.00%.The occurrence of treatment-related side effects was comparable in both the fasted and postprandial groups, as was the incidence of adverse drug reactions.

**Conclusion:**

The study concluded that the high-fat meal had a notable impact on how Ondansetron is processed in the body. Healthy subjects tolerated all treatments well and safely under both postprandial and fasted conditions.

**Clinical Trial Registration:**

http://www.chinadrugtrials.org.cn/index.html, identifier CTR20213116.

## Introduction

Cancer poses a worldwide health challenge, with chemotherapy being a primary form of treatment. Unfortunately, chemotherapy can result in various negative side effects, including chemotherapy-induced nausea and vomiting (CINV) (Jordan et al., 2015). Approximately 70%–80% of adult cancer patients suffer from CINV. Chemotherapy-induced nausea and vomiting (CINV) continues to be a significant issue linked to cancer treatment. It can result in electrolyte imbalances and malnutrition, as well as heighten feelings of anxiety and depression in patients, impacting their treatment compliance and potentially causing treatment delays or serious consequences (Piechotta et al., 2021; Grassi et al., 2015). Providing preventive measures for CINV is a crucial aspect of cancer care for numerous patients (Lyons et al., 2021).

Ondansetron, a novel carbazole compound, selectively and strongly blocks serotoninergic neurotransmission at serotonin 3 (5-HT3) receptors. Originally developed for treating chemotherapy-induced vomiting, it was introduced for human use in 1984 (Robak, 1993; Blackwell and Harding, 1989).

Ondansetron effectively treats sudden nausea and vomiting in patients undergoing high-intensity chemotherapy. Additionally, it is beneficial for patients who have not responded favorably to other anti-nausea medications after receiving less intense chemotherapy. Furthermore, Ondansetron is a viable option for those who experience intolerable side effects from traditional anti-nausea drugs. Lastly, it can also be used to manage nausea and vomiting in patients undergoing radiation therapy in the upper abdomen (Scotté et al., 2023; Razvi et al., 2019; Dennis et al., 2011; Feyer et al., 2011).

It is not only effective in treating chemotherapy-induced nausea and vomiting (CINV), but is also approved for preventing and treating postoperative nausea and vomiting (PONV) (Currow et al., 1995). Moreover, due to its effectiveness and minimal side effects, it is commonly used to treat and prevent nausea and vomiting in pregnant women and children (Patel et al., 2017; Smith, 1989; Masarwe et al., 2023; Kaplan et al., 2019). It is considered both beneficial for patients and cost-efficient (Weghorst et al., 2021).

Food can alter drug absorption in multiple ways, while formulation elements can also impact the interaction between food and drugs (Melander and McLean, 1983; Singh, 1999; Gu et al., 2007; Song et al., 2024).

Results of the studys showed that a slight increase in bioavailability of ondansetron with standard postprandial administration (Roila and Del Favero, 1995; Bozigian et al., 1994). Given that the aforementioned research focused on how regular meals affect the absorption of ondansetron, it is necessary to measure the influence of food consumption on the drug’s effectiveness to assess the practical significance of a food-drug interaction (Schmidt and Dalhoff, 2002).

The aim of this research can be expressed as exploring the impact of a high-fat meal on the pharmacokinetics of Ondansetron and assessing the safety of orally administering 8 mg of Ondansetron to healthy volunteers.

### Subjects

Approximately 10 days prior to drug administration, individuals in good health from China were evaluated for qualification. In order to participate, Chinese adults who are in good health must willingly provide informed consent. Both men and women who are at least 18 years old with a body mass index (BMI) between 19 and 26 kg/m2, and a minimum weight of 45 kg for females and 50 kg for males are eligible. The participants did not have any immediate plans for fertility, chose to use non-pharmacological contraceptive methods voluntarily, and did not intend to donate eggs or sperm. Additionally, there was no record of cardiovascular, neurological, metabolic, infectious, hepatic, neurological, pulmonary, endocrine, immunological, or psychiatric disorders among the participants. Participants with a background of alcoholism, smoking, or currently, or drug addiction taking medications that affect liver enzymes, or who had recent surgery were not included in the research. Subjects with abnormal vital signs, physical exam results, or significant laboratory findings were not included in the study. Those with allergies, especially to any component of Ondansetron or its excipients, special dietary needs, or lactose intolerance were also excluded. Additionally, pregnant women, those planning pregnancy, or using oral contraceptives were not part of the study. Taking additional medications was prohibited during the research. Volunteers were allowed to pull out of the study at their own discretion.

The study population was sourced from a bioequivalence clinical trial conducted at the drug clinical trial institution of Hebei General Hospital. This bioequivalence clinical trial was scheduled to commence on 24 October 2021, and it received approval for its clinical trial protocol and amendments from the Medical Ethics Committee of Hebei General Hospital on 6 November 2021, with approval number 2021-24-01.

The clinical trial for bioequivalence was registered on the Chinese Clinical Trial Registry website. (http://www.chinadrugtrials.org.cn/index.html #CTR20213116, date: 09 Dec 2021). The clinical trial will commence with a kick-off meeting on Dec. 06, 2021, followed by the first subject signing informed consent on Dec. 13, 2021.

The registration of drug trials in China is commonly used, but unfortunately, it is not recognized by the WHO. So we retrospectively registered our clinical trial in the China Clinical Trial Registration Center approved by the WHO. [https://www.chictr.org.cn/] (number: ChiCTR2400087981, date: 8 August 2024).

### Study design and procedures

Bioequivalence clinical trials were conducted in a randomized, open-label, single-dose, two-sequence, two-cycle, double-cross trial. Each period was followed by a 7-day washout period, and while maintaining confidentiality of all associated data, the plasma concentration analyses of ondansetron were performed.

The bioequivalence study included two separate trials: one conducted after fasting and the other after eating. The fasted group involved 26 participants who were divided into two groups, A (TR) and B (RT), each consisting of 13 subjects selected at random.28 subjects were enrolled in the postprandialeral group, with 14 participants randomly assigned to groups C (TR) and D (RT) each. The T and R formulations used in the study were ondansetron tablets manufactured by Shijiazhuang No. 4 Pharmaceutical Co. Ltd. and ondansetron tablets marketed as Zofran by Novartis Pharmaceuticals Corp.

This study aims to assess how food affects the absorption of ondansetron by comparing and analyzing the pharmacokinetic parameters of ondansetron in both fasted and postprandial trials.

This study analyzed the impact of food on ondansetron bioavailability by examining the pharmacokinetic parameters of preparation R in subjects who consumed it under both fasting and high-fat diet conditions. The fasted trials enrolled 26 subjects and all of subjects completed the test.

**FIGURE 1:**
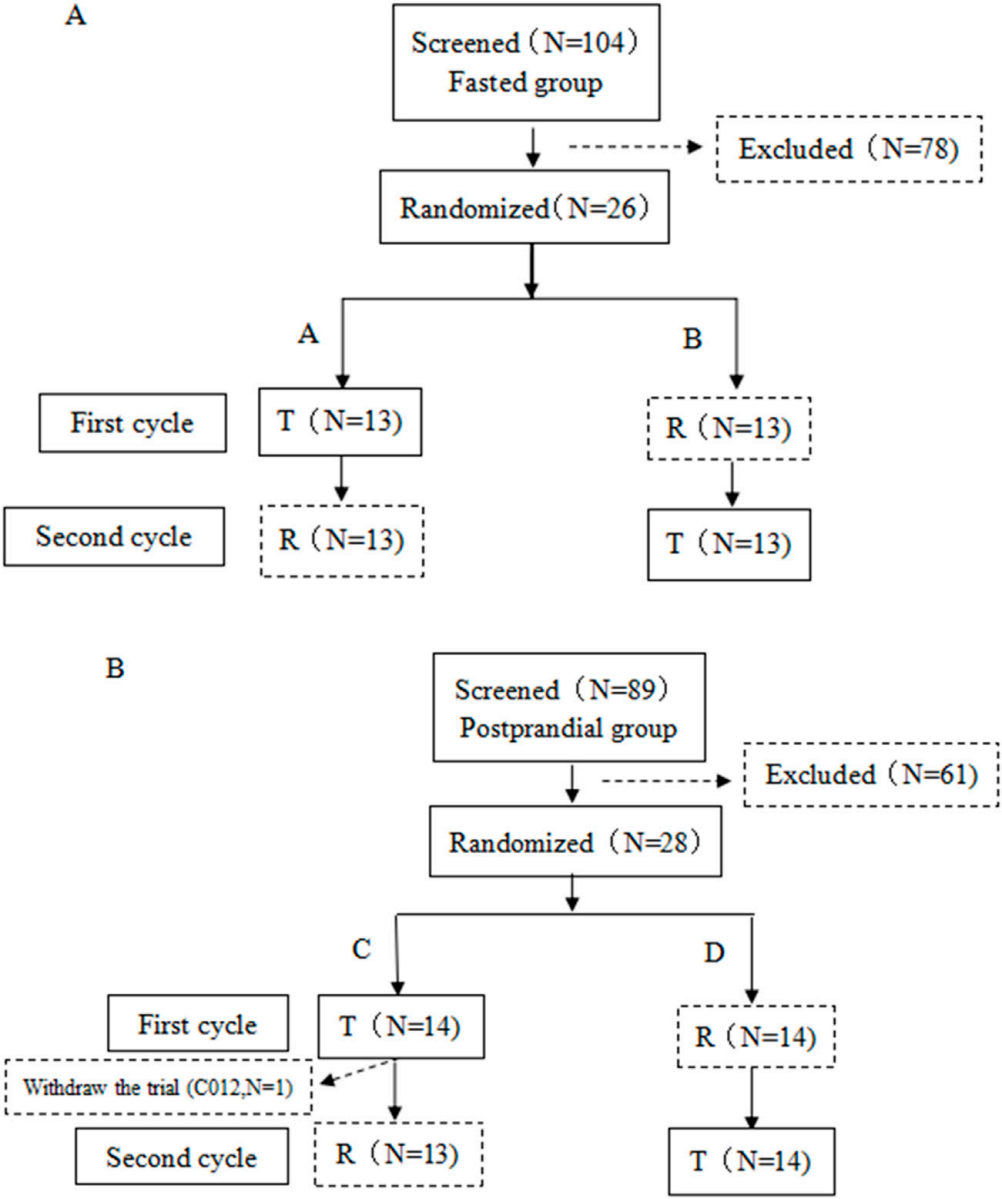
Subjects flow chart. **(A)** Flow chart of subjects in the fasting status. **(B)** Flow chart of subjects in the postprandial status. The quantity of subjects is represented by N.

The postprandial trials included 28 healthy volunteers, one of whom did not take the R preparation due to voluntary withdrawal from the test. Figure 1 illustrates the distribution of participants who were screened and ultimately included in the two groups.

Healthy volunteers were given a single oral dose of 8 mg ondansetron tablets from Novartis Pharmaceuticals Corp. (Germany, batch number 19M002) and their main pharmacokinetic parameter was analyzed in both fasted and postprandial conditions.

In the study, a total of 53 healthy volunteers were included in the trial, and the volunteers were divided into two following groups. The group that fasted received 8 mg ondansetron tablets after fasting for at least 10 h, while the group that ingested a meal with a high fat content was prescribed 8 mg ondansetron tablets, administered 30 minutes after the meal’s start. This meal, rich in fat, comprised between 800 and 1,000 kcal, with approximately 50% of its calories originating from fat, as outlined in Table 1.

**TABLE 1:** displays the ingredients needed for a diet high in fat.

Food variety	Total	Protein (Kcal)	Fat(Kcal)	Carbohydrate (Kcal)	Calories (Kcal)
Yili milk	250 mL	32.0	85.5	50.0	167.5
Yurun sausage	60 g	43.4	76.7	21.4	141.5
Eggs	110.00 g	45.5	88.8	7.9	142.2
Weichudao Croissant	115 g	34.0	283.6	209.8	527.4
calories (Kcal)		154.9	534.6	289.1	978.6
Calories ratio (%)		15.8	54.6	29.5	100

Notes: The energy content of 1 g of fat was determined to be 9 kcal, while 1 g of protein and 1 g of carbohydrate were both determined to be 4 kcal each. The energy content of eggs was calculated based on their raw form.

### Pharmacokinetic evaluations

Blood samples of subjects were collected for pharmacokinetic (PK) analysis. Blood samples from the fasting group was conducted before (within 60 min) and after administration at 0.25, 0.50, 0.75, 1.00, 1.33, 1.67, 2.00, 2.33, 2.67, 3.00, 4.00, 5.00, 6.00, 8.00, 10.00, 12.00 h, 24.00 h, with about 4 mL of blood collected at each point, and a total of 18 points were collected. The subjects in the postprandial administration test were treated at 0 h (after meal to before medication) and after medication 0.33, 0.67, 1.00, 1.33, 1.67, 2.00, 2.33, 2.67, 3.00, 3.50, 4.00, 5.00, 6.00, 8.00, 10.00,12.00 and 24.00 h, a total of 19 points were collected. The collected blood samples were placed in the pre-cooled labeled collection vessels, mixed upside-down for 4–8 times, centrifuged at 4°C for 10 min within 1 h (centrifugal force: 2500 g), and then separated the plasma samples. Plasma samples were kept in a refrigerator at −80°C within 2 h of being centrifuged. Ondansetron plasm levels were measured using liquid chromatography-mass spectrometry (LC-MS/MS).

### Safety evaluations

Evaluate safety with reference to CTCAE 5.0 standards and observe any abnormalities that occur in all subjects during the clinical study. Recording clinical characteristics, severity, timing of occurrence, duration, treatment methods, and outcomes included adverse reactions, severe adverse reactions, drug interactions, laboratory tests (blood tests, blood biochemistry, urine tests + urinary sediment analysis), blood pregnancy tests (for women only), electrocardiograms (ECGs), monitoring vital signs, physical examinations, and unexpected examinations. Assess its significance in relation to the investigational medication.

### Statistical analysis

The pharmacokinetic parameters of ondansetron after 8 mg single administration of Ondansetron tablets after fasting and high-fat diet were calculated by non-ating-room analysis method using Phoenix WinNonlin 8.1 software, and descriptive statistical analysis was performed AUC calculation was performed using the Linear Up-Log-Down trapezoidal approach.

WinNonLin’s BE module was used to analyze AUC_0–t_, AUC_0–∞_, and C_max_ of ondansetron under fasted and postprandial conditions after logarithmic conversion. The model estimated the adjusted mean difference (postprandial/Fasted) and its 90% confidence interval, which was then used to calculate the negative-valued number needed to determine the geometric mean ratio of the PK parameter comparing postprandial to fasted conditions. This ratio was used to estimate the corresponding 90%CI and assess the impact of a high-fat meal on ondansetron’s pharmacokinetics.

### Bioanalytical methods

The plasma concentration of ondansetron in healthy subjects was determined by LC-MS/MS method. A Shimadzu LC-20A from Japan and a Shimadzu LCMS-8045 mass spectrometer equipped with an ESI source were utilized. Initially, ondansetron-d3 was introduced as an internal standard, followed by the precipitation of protein from the plasma samples. Next, a volume of 5.0 μL of the supernatant was injected and separated on a WELCH ULTIMATE XB-C18 (2.1 × 100 mm, 5 μm) column using gradient elution at a flow rate of 0.6 mL/min (with mobile phase A containing 0.1% formic acid in 5 mM ammonium acetate and mobile phase B containing 0.1% formic acid in methyl alcohol). The temperature of the column was kept at 40°C. For quantification, two transitions were recorded: m/z 294.2/170.2 for ondansetron and m/z 298.2/174.1 for ondansetron-d3. The ideal settings for the instrument were established with the heater temperature set to 400°C and the ion spray voltage at 4000 V. Both ondansetron and ondansetron-d3 had a residence time of 150 ms. Ondansetron’s pre-four-pole deflection voltage deviation Q1 Pre was −22 V, collision energy (CE) was −27 eV, and pre-four-pole deflection voltage deviation Q3 Pre was −30 V. For Ondansetron-d3, Q1Pre was −12 V, CE was −27 eV, and Q3Pre was −17 V.

The intra-batch precision of all concentration control samples except LLOQ was no more than 7.0%, and the inter-batch precision was no more than 5.4%, The intra-batch precision of LLOQ was less than 10.9%, and the inter-batch precision was 8.8%; The intra-batch accuracy deviation of all concentration quality control samples except LLOQ ranges from −2.1% to 11.0%, and the inter-batch accuracy deviation from 2.3% to 5.8%, The intra-batch accuracy deviation of LLOQ ranges from −2.0% to 5.5%, and the inter-batch accuracy deviation was 2.5%, The precision and accuracy of ondansetron in plasma are shown in table 2.

**TABLE 2:** Precision and recovery of Ondansetron in plasma.

Concentration (ng·mL^−1^)	Intra -day			Inter -day			Absolute recovery (%, $\bar{x}\pm s$ )
	Measured (ng·mL^−1^, $\bar{x}\pm s$ )	RSD (%)	RE (%)	Measured (ng·mL^−1^, $\bar{x}\pm s$ )	RSD (%)	RE (%)	
0.20	0.21 ± 0.02	10.90	5.50	0.21 ± 002	8.80	2.50	-
0.60	0.62 ± 0.02	2.40	2.83	0.62 ± 0.03	5.40	2.33	102.14 ± 4.68
6.00	6.67 ± 0.25	3.70	11.02	6.35 ± 0.31	4.80	5.82	-
36.00	38.75 ± 0.70	1.80	7.64	37.06 ± 1.72	4.60	3.00	97.05 ± 2.94
90.00	96.73 ± 1.20	1.20	7.47	94.45 ± 2.60	2.70	4.94	104.51 ± 8.79

RSD: relative standard deviation; RE: relative error.

## Results

### Subjects

This study was conducted between October 2021 and March 2022. A total of 54 participants were asked to join the research project and were divided into either a group that had fasted or a group that had eaten. The fasted group consisted of 23 men and 3 women, while the postprandial group consisted of 24 men and 4 women. Table 3 displays the demographic and baseline traits of every participant. 53 subjects were recruited for a study evaluating the pharmacokinetic characteristics of ondansetron in fasting conditions and exploring the consequences of food intake on its pharmacokinetic behavior.

**TABLE 3:** Demographic baseline.

Variable	Fasted group	Postprandial group
Age(years)		
Mean ± SD	28.50 (8.20)	27.86 (6.15)
Median (Q1; Q3)	26 (22; 36)	27 (23; 31.5)
Min; Max	18; 45	19; 45
Sex n (%)		
Male	23 (88.46)	24 (85.71)
Female	3 (11.54)	4 (14.29)
Height (cm)		
Mean ± SD	170.27 (6.66)	171.11 (8.12)
Median (Q1; Q3)	171.5 (167; 175.5)	170.25 (167.5; 176.75)
Min; Max	155.5; 180.5	152.0; 192.5
Weight (kg)		
Mean (Std)	66.00 (8.41)	66.71 (7.74)
Median (Q1; Q3)	66.7 (59.4; 72.7)	66.1 (61.45; 72.9)
Min; Max	51.2; 80.5	55.1; 84.4
BMI(kg/m2)		
Mean (Std)	22.68 (1.77)	22.80 (2.30)
Median (Q1; Q3)	22.8 (21.1; 24)	23.5 (20.4; 24.85)
Min; Max	19.1; 26.0	19.1; 26.0

### Pharmacokinetic evaluations

**FIGURE 2:**
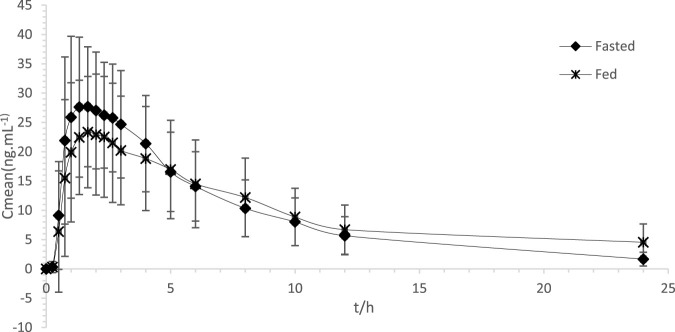
Comparison of average plasma concentrations over time for ondansetron following a single 8 mg oral dose of ondansetron tablet in both postprandial and fasted states.

Figure 2 displays the average plasma levels of ondansetron over time in healthy Chinese participants who were given a single 8 mg ondansetron tablet orally.

Table 4 displays the C_max_, AUC_0–t_, AU_C0–∞_, as well as additional pharmacokinetic endpoints (T_max_, t_1/2_) for ondansetron. The average C_max_, AUC_0–t_ and AUC_0–∞_ of ondansetron in plasma were lower after consuming a high-fat meal compared to fasting, with values of 28.57 ± 10.81 ng/mL, 181.74 ± 87.86 ng × h/mL, and 194.07 ± 98.79 ng × h/mL, respectively. The T_max_ and t_1/2_ of ondansetron in plasma following a high-fat meal were 1.79 ± 0.80 h and 5.71 ± 1.03 h, respectively, while under fasting conditions, they were 1.64 ± 0.81 h and 5.85 ± 0.99 h.

**TABLE 4:** Pharmacokinetic data for Ondansetron tablets after a 200 mg oral dose in healthy participants are shown.

PK parameter	Arithmetic Mean (SD)		Geometric Mean (CV(%))		LSGM ratio postprandial/fasted (90%CI)
	Fasted	Postprandial	Fasted	Postprandial	
*C*_max_ (ng/mL)	32.91 (10.60)	28.57 (10.81)	31.47 (30.7)	26.52 (42.4)	86.81% (71.48% - 99.39%)
AUC_0-t_ (ng × h/mL)	222.04 (93.57)	181.74 (87.86)	204.36 (43.9)	163.59 (49.8)	81.85% (65.17% - 98.30%)
AUC_0-∞_(ng × h/mL)	237.40 (105.28)	194.07 (98.79)	216.70 (46.2)	173.05 (52.2)	81.75% (64.42% - 98.95%)
_Tmax_(h)	1.64 (0.81)	1.79 (0.80)	-	-	-
*t*_1/2_(h)	5.85 (0.99)	5.71 (1.03)	5.76 (17.3)	5.61 (19.0)	-

90%CI: 90% confidence interval; Compared with the fasted group, P < 0.01.

### Effect of a high-fat meal on the pharmacokinetics

Table 4 displays the least-squares geometric mean ratio (LSGM) and its 90% confidence interval (CI) for ondansetron under both fasted and postprandial conditions.

After consuming a high-fat meal, the levels of ondansetron in plasma were reduced by 13.19% for C_max_, 18.15% for AUC_0–t_, and 18.25% for AUC_0–∞_ compared to when taken on an empty stomach. The 90% confidence intervals for C_max_, AUC_0–t_, and AUC_0–∞_,under fasted and postprandial conditions ranged from 71.48 to 99.39, 65.17 to 98.30, and 65.17 to 98.30, respectively, all falling outside the acceptable range of 80.00%–125.00%. The presence of a high-fat meal significantly impacted the pharmacokinetics of ondansetron, although T_max_ and t_1/2_ remained unaffected by the meal. Since T_max_ and t_1/2_ were not impacted by food consumption, the variations in ondansetron exposure between postprandial and fasted states were probably due to reduced bioavailability in the postprandial state.

The reference preparation used in the bioequivalence test was the source of the data analyzed in this study. During the bioequivalence assessment, the test and reference preparations showed bioequivalence when tested and fasting conditions. The C_max_, AUC_0-t_ and AUC_0-∞_ of the experimental sample were 29.78 ± 9.28 ng/mL, 203.08 ± 86.82 h·ng/mL and 216.30 ± 95.69 h·ng/mL, respectively. During the post-meal examination, it was determined that the test and reference preparations had similar biological effects. The C_max_, AUC_0-t_ and AUC_0-∞_ of the experimental sample were 27.66 ± 11.72 ng/mL, 171.46 ± 93.52 h·ng/mL, and 183.20 ± 105.87 h·ng/mL.

### Safety evaluations

In healthy participants, Ondansetron 8 mg was well tolerated under both postprandial and fasted conditions, with no adverse events or serious adverse events leading to the discontinuation of the drug observed in this study. Among the fasted participants, there were 6 instances of adverse events, accounting for 23.08%, and none were attributed to the medication being studied. Among those who consumed the high-fat meal, 4 instances of adverse events were reported, accounting for 14.81% of the group, with none being attributed to the investigational drug. The AEs were mild laboratory abnormalities (elevated fasting blood glucose, HGB decreased, scr elevated and total bilirubin elevation, direct bilirubin elevation, CK-MB increases, serum magnesium decreased, TG elevated). All mild adverse events were transient and possibly not related to the study drug; no participants stopped or left the trial because of adverse events. In general, there were no notable variations in negative occurrences between the high-fat meal group and the fasting group.

## Discussion

The impact of food on medication is a multifaceted relationship influenced by various factors such as the composition of the food, the patient’s physiological response to eating, the characteristics of the medication, and how it is taken. While there could be interactions between certain medications and certain foods, these interactions may not impact all medications or all individuals. Additionally, even if interactions do occur, they may not be clinically significant (Deng et al., 2017).

In most cases, the influence of food on the absorption of a drug is assessed based on the specific dietary factors that are anticipated to significantly alter the drug’s metabolism. The FDA recommends using high-fat meals (comprising approximately half of the total calories in a meal) as the standard for studying how diet affects pharmacokinetics, in order to maximize the impact of food. In this study, the total calorie content of the high-fat diet was 978.60 kcal, with fat accounting for 54.60% of the total calories, and 534.60 kcal came from fat.

Ondansetron is a commonly used antiemetic drug in clinical practice. Like all other medications taken by mouth, it is crucial to assess how food impacts its absorption in order to give recommendations on when to take it with meals. At present, it remains uncertain if the pharmacokinetic profile of 8 mg ondansetron tablets in the Chinese population would be impacted by a high-fat meals. The impact of a high-fat meal on the pharmacokinetics of orally ingested ondansetron tablets (8 mg) was assessed in this research. The findings indicated that the presence of a high-fat meal while taking ondansetron led to a reduction of 13.19% in C_max_, 18.15% in AUC_0-t_, and 18.25% in AUC_0-∞_.The 90% confidence intervals (CI) for C_max_, AUC_0-t,_ and AUC_0-∞_ under fasting and postprandial conditions were 71.48 ∼ 99.39%, 65.17 ∼ 98.30%, and 65.17 ∼ 98.30%, respectively, none of which fell within the range of 80.00 ∼ 125.00%.There were varying degrees of delay and shortening in T_max_ and t_1/2_, but these changes were not statistically significant (P > 0.05). The study findings suggest that giving ondansetron with a high-fat meal can greatly decrease its absorption rate.

In this study, the pharmacokinetic characteristics of ondansetron in healthy volunteers showed no significant differences compared to those reported in the literature (Cánovas et al., 2012; Jiang et al., 2016; Di et al., 2011). However, some literature reports indicate that the bioavailability of 8 mg ondansetron tablets taken after a standard meal is slightly higher than that of fasting subjects (Roila and Del Favero, 1995; Bozigian et al., 1994).

These research results suggest that the level of fat content in the diet can affect the pharmacokinetic parameters of ondansetron.

Fatty foods can greatly slow down the emptying of the stomach in comparison to a diet low in fat (Stacher et al., 1991). This can lead to an increase in bile secretion, which can have a multifaceted impact on the absorption of oral medications (Yeap et al., 2013). The increased bile secretion can improve the absorption of lipophilic drugs by aiding in their solubilization and by enhancing drug absorption through the lymphatic system in the intestines. Nevertheless, it also inhibits epithelial transporters, resulting in a different intricate impact on the absorption of oral medications. Moreover, consuming fatty foods can lead to gastrointestinal discomfort, like diarrhea, that can hinder the absorption of oral medications. This kind of diet slows down the emptying of the stomach, prolongs the presence of ondansetron in the gastric cavity, and diminishes its absorption rate in the small intestine.

No significant variance was observed in the occurrence of adverse events (AEs) between the group that fasted and the group that ate after the meal. Severity of all adverse events was classified as grade 1 and they were potentially not associated with the investigational drug. They were transient and resolved spontaneously without any intervention. No serious adverse events (SAEs) occurred in either group. This suggests that ondansetron is well-tolerated and safe in both fasting and postprandial states, which aligns with existing literature (Baber et al., 1992).

There are several limitations in our study. Initially, our research focused solely on examining the pharmacokinetic properties of a solitary oral ondansetron tablet when consumed with a high-fat diet. Additionally, the pharmacokinetic characteristics of ondansetron were not investigated in individuals experiencing nausea and vomiting and following a high-fat diet, potentially showing variations compared to those in individuals without health issues. Hence, we will investigate how different diets affect the way ondansetron is processed in patients experiencing nausea and vomiting from various causes, particularly focusing on how multiple doses of ondansetron impact its pharmacokinetic properties.

## Conclusion

In Chinese subjects, a high-fat diet significantly decreases the exposure of the 8 mg therapeutic dose of ondansetron. Healthy volunteers who received a single oral administration of 8 mg ondansetron demonstrated good safety and tolerability in both fasting and postprandial states.

## Data Availability

The original contributions presented in the study are included in the article/supplementary material, further inquiries can be directed to the corresponding author.
